# Potential of Health and Demographic Surveillance System in Asthma and Chronic Obstructive Pulmonary Disease Microbiome Research

**DOI:** 10.3389/fpubh.2017.00196

**Published:** 2017-08-04

**Authors:** Dhiraj Agarwal, Dhiraj Dhotre, Rutuja Patil, Yogesh Shouche, Sanjay Juvekar, Sundeep Salvi

**Affiliations:** ^1^Chest Research Foundation, Pune, India; ^2^Vadu Rural Health Program, KEM Hospital Research Centre, Pune, India; ^3^Microbial Culture Collection, National Centre for Cell Science, Pune, India; ^4^INDEPTH Network, Accra, Ghana

**Keywords:** asthma, chronic obstructive pulmonary disease, health and demographic surveillance system, microbiome, microbiota

## Abstract

Health and demographic surveillance system (HDSS) is a population-based health and vital event registration system that monitors demographic and health events in a geographically defined population at regular intervals. Human microbiome research in the past decade has been the field of increasingly intense research much due to its demonstrated impact upon various health conditions including human chronic airway diseases such as asthma and chronic obstructive pulmonary disease (COPD). Many confounding factors have been revealed to play a role in shaping the microbiome in chronic airway diseases. Asthma and COPD follows a typical pattern of disease progression, which includes stable and exacerbation state in which the microbiota is known to vary. However, many such studies lack extensive and longitudinal sampling with inadequate metadata, which has resulted in the inconsistencies in the observations. HDSS provides such a platform, which can offer a deeper understanding of the role of the microbiome in human health. In this review, we highlight opportunities and limitations in microbiome research with the help of studies conducted on chronic airway diseases like asthma and COPD. In addition, we also emphasize on the benefits of HDSS and future directions in lung microbiome research.

## Introduction

Health and demographic surveillance system (HDSS) is a population-based health and vital event registration system that collects and monitors demographic and health events information in a geographically defined population at regular intervals (1). HDSS is usually comprised of a large team of qualified professionals and volunteers involved in large-scale data collection, monitoring, analysis, and report generation in a specific population for a long period of time. HDSS sites, because of their reliable information, can help policy makers to prioritize health issues and accordingly allocate resources more efficiently. It can act as a platform for intervention studies and provides feedback on programs’ effectiveness, which consequently helps in improving policies (2). INDEPTH is a global network of HDSS sites comprising of 42 member centers, which observe through 47 HDSS field sites (http://www.indepth-network.org/). These sites monitor life events of over three million individuals in 18 low and middle-income countries from Africa, Asia, and Oceania (3). Unlike cohort studies, HDSS follows the entire population in a defined geographic area for an ongoing period of time. During its initial census, HDSS registers all households and individuals who are then followed by frequent documentation of events such as births, deaths, marriages, and migrations in subsequent census rounds. Furthermore, information pertaining to health, social, and economic aspects of the population under surveillance is also collected, which eventually serves as a comprehensive metadata for any study on human populations. As a result, HDSS provides a platform for various research activities and offers a ready-made sampling frame for tracking the population. Apart from demographic information, any other information relevant to the particular study can be easily augmented and linked with other datasets in HDSS program (4).

We are increasingly able to understand that microbes contribute in many aspects of human health and physiology. Many intrinsic and extrinsic factors like mode of birth, infant feeding patterns, antibiotic usage, sanitary habits, age, genetics, diet, geography, socioeconomic status, and disease condition have been shown to alter the microbiome, which in turn affects human health (5–9). Extensive studies have been conducted across the globe focusing on the influence of microbiome in chronic diseases like asthma, chronic obstructive pulmonary disease (COPD), inflammatory bowel disease, obesity, diabetes, and cardiovascular diseases (10–15). Apart from genetics, many environmental factors are known to be associated with these diseases (16). Based on recent studies of the microbiome, it is clear that a robust research framework accompanied by a collection of comprehensive metadata is essential in building our knowledge of this tripartite relationship between humans, environment, and the microbes.

Asthma and COPD are common chronic airway diseases with high prevalence and mortality rates across the world (17, 18). Although several studies have linked various factors such as genetic predisposition, smoking, air pollution, and occupational exposures to these diseases; microbes are also known to play a vital role (19–22). In the past decade, the discovery of the human respiratory tract microbiome has steered growing number of studies to understand the role of the microbiome in asthma and COPD (23). Understanding why and how these diseases develop and progress over time requires long-term follow-up. Many cohorts have been established worldwide, such as ECLIPSE, CanCOLD, Birmingham, MicroCOPD (24–27). Although advancements have been made regarding descriptions of the main characteristics of chronic airway diseases, much is still to be explored about the factors associated with changes in the microbiome and the relevance of these changes in host-specific pathogenesis of the disease (23).

In this review, we discuss microbiome studies on chronic airway diseases like asthma and COPD to understand how HDSS can aid in human microbiome research. In addition to this, we also highlight opportunities and limitations in human microbiome research and also show how HDSS can benefit this field in future.

## Microbiome in Asthma

Many factors, both environmental and genetic are responsible for the onset of asthma but no single cause has been identified so far. Although many studies including a study by Hilty et al. suggests a role for the microbiome in the etiology of asthma (28), the association is still not clearly understood (29). Evidence of colonization or infection with specific microbial species has been linked with the development or presence of asthma (30). The existence of abundant and diverse microbiota in airways suggests that they can modulate airway function and immunological responses (31, 32). In this context, members of phylum *Proteobacteria* like *Haemophilus* spp., *Tropheryma whipplei, Moraxella catarrhalis*, and *Neisseria* spp. have shown a strong correlation between the presence of asthma and its exacerbations (28, 33, 34). Genus *Pseudomonas* and *Lactobacillus* of phylum *Proteobacteria* and *Firmicutes*, respectively, dominate the oropharynx in asthmatics (35).

**Figure 1:**
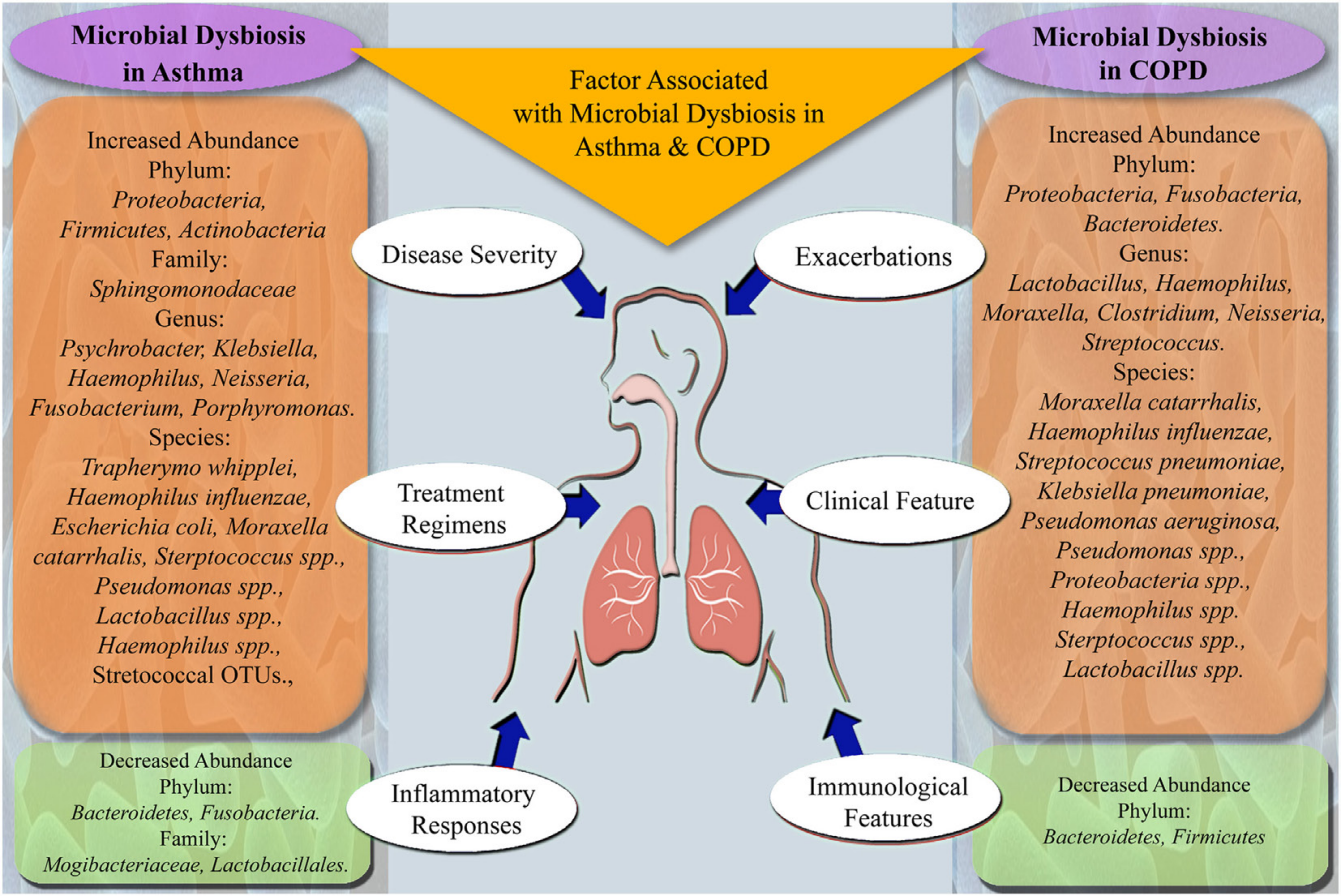
Summary of microbes altered in microbial dysbiosis and factors associated with microbial dysbiosis in asthma and chronic obstructive pulmonary disease (COPD) subjects.

Recently, it has been reported that specific bacteria are associated with different stages of asthma severity and phenotypes (33, 36, 37). Castro-Nallar et al. and Depner et al. have shown that nasal microbial communities are different in asthmatic children compared to healthy children and pathogenic *M. catarrhalis* are present in abundance in asthmatic children (38, 39). Similarly, Marri et al. found higher diversity among microbial communities in asthma compared to healthy with *Proteobacteria* being the most abundant phyla but showed similar microbial composition in mild and severe asthma (40). Another study in asthma and COPD have shown the statistically non-significant difference in overall airway microbiota between two groups. But they found that *Pseudomonas* spp. and *Lactobacillus* spp. were highly abundant in both groups (35). Zhang et al. have shown that the presence of *Streptococcus* spp. in the airway is associated with severe asthma (41). Huang et al. revealed that specific bacteria are associated with asthma severity and observed airway microbial dysbiosis in subjects with severe asthma as compared to mild asthma (37). Summary of bacterial groups found to be altered and factors known to be associated with microbial dysbiosis in asthma subjects is given in Figure 1.

In spite of the relatively large body of epidemiologic literature on the association of microbiome and asthma, the evidence overall remains inconclusive (19). It was learned from above studies that it is difficult to discriminate between resident and transient microbes, hence studies discussed here do not give the real picture of the microbes associated with a disease condition. This could be because many intrinsic and extrinsic factors are known to play a key role in shaping the microbiome, which necessitates collection and analysis of relevant metadata to understand their effect on the microbiome.

## Microbiome in COPD

Various risk factors are associated with COPD, including tobacco smoke, household air pollution, occupational exposure, etc. However, the role of microbial infection in COPD exacerbations is debated (29). Like asthma, the role of the microbiome in COPD was first shown by Hilty et al. (28). Among the most studied phylum, *Proteobacteria, Haemophilus* spp. are frequently found in bronchi of COPD subjects and members of phylum *Bacteroidetes* are more commonly found in healthy airways (42, 43). Previous studies do not suggest any correlation of conventionally known bacterial pathogens with COPD exacerbations. However, a study by Aguirre et al. identified pathogenic genus *Haemophilus* and *Moraxella* in COPD, which could not be detected previously by culture-dependent approach (44).

Erb-downward et al. suggested lower microbial diversity in patients with decreased lung function (45). Similarly, Garcia-Nuñez et al. showed that decrease in microbial diversity is associated with disease severity in COPD (46). On the contrary, Pragman et al. indicated that there is a significant increase in microbial diversity with the development of COPD (47). The latest study also mentioned that age is associated with increased microbial diversity in COPD, unlike the previous studies where age factor was not considered. Erb-downward et al. showed that in never-smokers, smokers, and COPD, microbiota is similar; however, it differs at microanatomic sites in lungs (45). Similarly, Cabrera-Rubio et al. showed the existence of different microbiota in the upper and lower compartments of the respiratory tract in COPD subjects (48). Pragman et al. revealed that anaerobic bacteria from the phylum *Fusobacteria* and *Bacteroidetes* and genus *Clostridium* are present in the air-rich environment of lungs of COPD subjects (47). Zakharkina et al. showed the varying prevalence of various genera such as *Moraxella, Neisseria*, etc., in COPD and healthy individuals (49). Sze et al. mentioned that there is a detectable bacterial community within lung tissue, which is different in subjects with very severe COPD as compared to healthy (50). Wu et al. showed a significant increase in abundance of *Streptococcus pneumoniae, Klebsiella pneumoniae*, and *Pseudomonas aeruginosa* in COPD as compared to healthy (51). Recently, this was further confirmed by Garcia-Nuñez et al. that potentially pathogenic microorganisms other than *P. aeruginosa* can be the cause of exacerbations in COPD, and hence antibiotic treatments against the activity of other pathogens need to be considered while treating COPD (52).

In addition to microbial profiling, a study by Lee et al. on metatranscriptomic profiles in COPD patients showed inconsistencies with the results obtained by microbial profiling (53). Furthermore, functional metagenomics of lower and upper respiratory tract microbiome in COPD patients showed the role of the microbiome in disease pathogenesis (54, 55). Studies have shown that exacerbations of COPD are associated with changes in the microbiome with an increase in specific taxa, which are related to typical COPD pathogens and decrease in microbes that contribute to compositional and functional homeostasis (56–58). Figure 1 summarizes the microbial taxa and factors found to be associated with microbial dysbiosis in COPD subjects.

Although adequate research has been carried out in COPD as compared to asthma, these studies also lack consistency in the results. Along with longitudinal studies and inclusion of comprehensive metadata, studies on genetically related and unrelated populations are also essential in these diseases as genetic factors have also shown to play a role in the development of COPD (22). Although disease-specific cohorts are available in COPD, no comprehensive longitudinal study considering genetic factors and other related metadata is available.

## Current Methodology for Lung Microbiome Research

Along with the culture-based techniques, many other approaches such as cloning, DGGE, PhyloChip microarrays, and T-RFLP were used to study the microbial communities in the human body (32, 37, 38, 42, 49–51, 57, 59). However, in the past decade, the field of microbial identification has changed enormously due to advancement in metagenomics research with the substantial support of the sequencing technologies. The majority of the studies conducted in asthma and COPD population explored microbiota by amplifying 16S rRNA gene by PCR followed by high throughput sequencing (10, 11, 33–36, 40, 43–48, 52, 55, 56, 60–62). These studies provided profiling of microbiota in the diseased condition. However, a study also performed predictive functional analysis based on the 16S rRNA gene sequences (63). In some studies, the bacterial burden was quantified with quantitative PCR based on the 16S rRNA gene (34, 36, 50, 51, 60, 64). Metagenomics studies involving transcriptomic and metatranscriptomic of both host and microbiota were also performed to understand the functional changes associated with asthma and COPD development (31, 39, 53–55, 58). These modern techniques have advanced the human microbiome research substantially.

## Opportunities and Limitations in Microbiome Studies for Asthma and COPD

The human microbiome research has expounded the important role of microbes in chronic diseases, including infectious diseases by exploring the functional capacities of microbes. It is clear from the previous studies that many factors are associated with changes in the microbiome like the severity of the disease, age, sampling site and size, treatment type, and antibiotic usage. Together with a disease condition, temporal studies considering these factors will help in better understanding of changes in microbial communities in asthma and COPD. Along with this, many other unexplored factors like occupational hazards, geographical location, hygiene and sanitation, social habits, and dietary habits also have a role to play in shaping the microbiome. Proper gathering of information of all the confounders associated with these chronic diseases is necessary to get deeper insights in human microbiome, but this is often a challenge because of lack of robust metadata and set up for longitudinal follow-up of the population.

Asthma and COPD follows a typical pattern of disease progression, which includes stable and exacerbation state in which the microbiota is also shown to vary. Hence, proper documentation and sample collection of the same patients at different stages of progression of the disease is necessary to understand the role of the microbiome in the disease condition. There is a need to re-examine the differences in the pattern of microbiota; before, during, and after the onset of exacerbations with detailed documentation of other important factors. This necessitates long-term follow-up of the population under study, which is a challenging task in the field of human microbiome research due to the paucity of comprehensive metadata and system for longitudinal follow-up of the population. The treatment regime of patients also alters the microbiota and many studies conducted so far lack the information on the use of antibiotics and medication. A study with COPD diagnosed patients, with and without any treatment may help in better understanding of the disease progression and role of microbes.

Although alterations in microbial communities are associated with severity of the disease, it is not clear whether they have a causative or exacerbating effect. This gives a unique opportunity to explore these associations with studies focusing on the establishment and development of microbiome and their role in normal functioning of the airway. This would eventually lead us to understand the succession and inoculation of microbiota in normal to the diseased condition. The understanding of the transient or permanent existence of microbiota in specific locations within airway would also lead to more information on the chronic nature of airway diseases. However, studies of short- and long-term antibiotic usage and its effect on the composition of microbiota in the context of airway microbiota need more attention.

Even though it is known that host’s genetic predisposition plays a role, many microbiome studies have not considered this factor. Proper control samples with comprehensive metadata from first-degree relatives of patients can help in exploring the effect of genetics in these chronic diseases. Moreover, studies on airway microbiome conducted so far include samples from genetically homogenous populations originating from the same geographic zone. As genetics is known a key factor in these diseases, similar studies on different populations are required to give a holistic picture. It is worth conducting such studies on a heterogeneous population with different ethnicity, heterogeneous diet, lifestyle, and climatic/geographic variation.

Most of the studies on asthma and COPD in this review lack extensive and longitudinal sampling with inadequate metadata collection. One of the reasons for this could be that these studies were either hospital-based or a part of related observational studies (Tables 1 and 2). There is a need to design broader microbiome research in asthma and COPD population with long-term follow-up, comprehensive metadata, and statistically significant sampling size. This will help in improving our knowledge of pathophysiology of airway diseases. This will also open the door for prebiotics, probiotics, or new antibiotics treatment in chronic airway disease management. This profiling of microbes in subjects with chronic airway diseases would also help physicians for diagnostic purpose with the help of advanced molecular tools of microbiome research. Some other reasons for inconsistencies in the results might be because of technical pitfalls (65). These factors are not discussed here as they are out of the scope of this review.

**Table 1:** List of microbiome studies conducted in asthma population.

Sr. no.	Reference	Year	Sample type	Sample size and study group	Sample Selection	Key findings
1	Castro-Nallar et al. (39)	2015	Nasal epithelial cells	8 asthma, 6 control	Cohort	Microbial communities differ in asthmatic as compared to healthy. The pathogenic *Moraxella catarrhalis* more dominant in asthmatic

2	Huang et al. (37)	2015	Bronchial brushings	40 severe asthma, 41 mild to moderate asthma, 7 healthy	Previous study cohort	Specific microbiota is associated with and may modulate inflammatory processes in patients with severe asthma and related phenotypes. More airway dysbiosis in severe asthma as compared to mild asthma

3	Zhang et al. (41)	2012	Bronchial brush, lavage cells, and sputum	29 severe asthma, 24 non-severe asthma, 19 healthy	Not mentioned	*Streptococcus* species is associated with severe asthma.

4	Park et al. (35)	2014	Oropharyngeal swab	18 COPD, 17 asthma, 12 controls	Not mentioned	No significant difference between microbiota in asthmatic and chronic obstructive pulmonary disease (COPD)

5	Marri et al. (40)	2012	Sputum	10 asthma, 10 controls	Previous study cohort	Asthmatics with greater diverse microbes as compared to healthy with the *Proteobacteria* being the most abundant phyla

6	Hilty et al. (28)	2010	Nose and oropharynx swabs	5 COPD, 11 asthma, 8 control, 13 asthma (pediatric), 7 controls	Not Mentioned	Pathogenic *Proteobacteria*, particularly *Haemophilus* species were much more frequent in asthmatics and COPD than controls

7	Zhang et al. (10)	2016	Sputum	26 severe asthma, 18 non-severe asthma, 12 healthy	Clinic-based recruitment	Sputum microbiota in severe asthma differs from healthy controls and non-severe asthmatics and is characterized by the presence of *Streptococcus* spp. with eosinophilia

8	Pérez-Losada et al. (31)	2015	Nasal epithelial cells	8 asthma, 6 healthy	Cohort	Enrichment analysis of 499 differentially expressed host genes for inflammatory and immune responses revealed 43 upstream regulators differentially activated in asthma. Microbial adhesion (virulence) and *Proteobacteria* abundance were significantly associated with variation in the expression of the upstream regulator IL1A

9	Huang et al. (32)	2011	Bronchial epithelial brushings	65 asthma, 10 healthy	Previous study cohort	The composition of bronchial airway microbiota is associated with the degree of bronchial hyperresponsiveness among patients with suboptimally controlled asthma

10	Durack et al. (33)	2016	Oral wash and bronchial brushings	42 steroid-naive atopic asthma, 21 with atopy but no asthma, 21 non-atopic healthy	Previous study cohort	Differences in the bronchial microbiome are associated with immunologic and clinical features of the disease

11	Simpson et al. (34)	2015	Sputum	46 asthma	Previous study cohort	Phenotype-specific alterations to the airway microbiome in asthma

12	Taylor et al. (36)	2017	Sputum	167 asthma	Previous study cohort	Neutrophilic asthma is associated with airway microbiology that is significantly different from that seen in patients with other inflammatory phenotypes, particularly eosinophilic asthma

**Table 2:** List of microbiome studies conducted in chronic obstructive pulmonary disease (COPD) population.

Sr. no.	Reference	Year	Sample type	Sample size and study group	Sample selection	Key findings
1	Sze et al. (43)	2015	Lung tissue	5 COPD, 4 control	Not mentioned	Host immune response to microorganisms within the lung microbiome contribute to the pathogenesis of COPD

2	Aguirre et al. (44)	2015	Sputum	19 COPD	Clinic-based recruitment	Found pathogenic genus *Haemophilus* and *Moraxella* in COPD, which was not previously diagnosed by culture method

3	Garcia-Nuñez et al. (46)	2014	Sputum	17 COPD (severity)	Clinic-based recruitment	As the severity of COPD increases, microbial diversity decreases

4	Park et al. (35)	2014	Oropharyngeal swab	18 COPD, 17 asthma, 12 controls	Not mentioned	No significant difference between microbiota in asthmatic and COPD

5	Zakharkina et al. (49)	2013	Broncho alveolar lavage	9 COPD, 9 healthy	Not mentioned	The presence of highly diverse bacterial communities in the lungs of healthy individuals and COPD patients

6	Pragman et al. (47)	2012	Broncho alveolar lavage fluid (BALF)	22 COPD, 10 controls	Previous study cohort	Increase in microbial diversity in COPD patients

7	Sze et al. (50)	2012	Lung tissue	8 non-smoker, 8 smoker, 8 COPD, 8 cystic fibrosis	Not mentioned	Lung tissue harbors detectable bacterial community and its changes in patients with severe COPD

8	Erb-downward et al. (45)	2011	BALF and lung tissue	3 never-smokers, 7 smokers, 4 COPD	Previous study cohort	Lesser microbial diversity in the patients with decreased lung function

9	Huang et al. (42)	2010	Endotracheal aspirates	8 COPD	Hospital database	Bacterial community diversity in COPD airways is substantially greater than previously recognized and includes a number of potential pathogens detected in the setting of antibiotic exposure

10	Hilty et al. (28)	2010	Nose and oropharynx swabs	5 COPD, 11 asthma, 8 control, 13 asthma (pediatric), 7 controls	Not mentioned	Pathogenic *Proteobacteria*, particularly *Haemophilus* species were much more frequent in asthmatics and COPD than controls

11	Wu et al. (51)	2014	Sputum	10 COPD, 10 healthy	Not mentioned	Significant increases of *Streptococcus pneumoniae, Klebsiella pneumoniae*, and *Pseudomonas aeruginosa* (*P* < 0.05) in the COPD group compared with the healthy group

12	Einarsson et al. (11)	2016	Bronchial wash	18 COPD, 8 smokers with no airways disease, 11 healthy	Clinic-based recruitment	Microbial community differs significantly in COPD in comparison with smokers and non-smokers. *Pseudomonas* spp. was greater in the lower airways of patients with COPD; however, the members of *Bacteroidetes*, such as *Prevotella* spp., were observed to be greater in the “healthy” comparison groups

13	Cabrera-Rubio et al. (48)	2012	Sputum, bronchial aspirate, bronchoalveolar lavage, and bronchial mucosa	6 moderate COPD	Previous study cohort	Bronchial tree is not sterile in COPD patients and different microbiota present in the upper and lower compartments of the respiratory tract

14	Garcia-Nuñez et al. (52)	2017	Sputum	21 COPD	Hospital-based cohort	The bronchial microbiome shows differences according to with *P. aeruginosa* biofilm-forming capacity

15	Lee et al. (53)	2016	Sputum	4 moderate, 4 severe COPD	Not mentioned	Bacterial composition determined by 16S rRNA gene sequencing may not directly translate to the set of actively expressing bacteria as defined by transcriptome sequencing

16	Cameron et al. (54)	2016	Sputum	8 COPD, 10 healthy	Previous observational study	Significant changes in the abundance of *Streptococcus* genus. The functional capacity of the COPD UBT microbiome indicated an increased capacity for bacterial growth

17	Wang et al. (56)	2016	Sputum	87 COPD	Not mentioned	Dynamic lung microbiota associated with exacerbation events and indicative of specific exacerbation phenotypes. Antibiotic and steroid treatments have differential effects on the lung microbiome

18	Millares et al. (55)	2015	Sputum	8 Severe COPD	Hospital-based cohort	Bronchial microbiome as a whole is not significantly modified when exacerbation symptoms appear in severe COPD patients, but its functional metabolic capabilities show significant changes in several pathways

19	Huang et al. (57)	2014	Sputum	12 COPD	12 COPD	Changes in the bacterial composition after treatment for an exacerbation differed significantly among the therapy regimens

20	Huang and Boushey (58)	2015	Sputum	12 COPD	12 COPD	Exacerbations of COPD are associated with heterogeneous changes in the bronchial microbiome, with increases in the abundance of species related to typical COPD pathogens and decreases in microbiota members that contribute to compositional and functional homeostasis

## How HDSS Can Benefit Microbiome Studies

**Figure 2:**
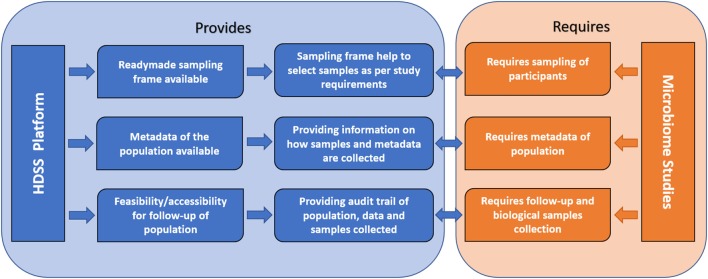
Conceptual framework showing how health and demographic surveillance system (HDSS) benefit human microbiome studies.

Inclusion and documentation of all the mentioned perplexing factors, which are known to alter the microbiome in chronic diseases can be achieved in the HDSS environment. HDSS can play an important role in risk factor surveillance and provide health information, which reflects the prevailing disease burden on populations more accurately. This would enable us to identify specific causes and their impacts on microbial composition during different phases of the disease. Many hurdles so far experienced in microbiome research, such as statistically significant sample size, long-term follow-up, and collection of comprehensive metadata can be overcome with HDSS setup. In microbiome research, it would be advantageous to collect maximum metadata on various confounding factors to interpret the data more accurately (9). For example, data on exposure to biomass and tobacco smoke, dust, pesticides, particulate matter, and other occupational or geographic factors may help in more accurate data interpretation in asthma and COPD rather than focus only on directly related factors such as disease condition and its severity. Such factors are shown to be a potential confounder in microbiome studies and controlling these factors in study design will help to increase reproducibility of results in future microbiome studies (9). HDSS provides a framework, which may consist of such information for various time points, thus, strengthening the metadata required for conducting human microbiome studies. HDSS provides an appropriate sample frame as the population covered under HDSS is longitudinally followed up and information on aspects like age, gender, and other sociodemographic characters is readily available (Figure 2).

Microbiome research on chronic diseases with known genetic basis requires comparison within and between distinct homogenous populations. The homogenous population, followed by HDSS in a defined geographic area would be consistent with environmental conditions and may also have a similar diet. As HDSS helps to generate a baseline data for comparison with the case of future studies, it is relatively easy to identify such prospective cohorts within HDSS population. This would help in addressing the issues of adjusting confounding factors for a particular microbiome study. Along with this appropriate control samples with comparable metadata for genetically related individuals of the patients can help in exploring the effect of genetics on microbiome in these chronic diseases.

In HDSS setup, after the provision for initial sampling, additional metadata and assessment of intervention can be added at any stage of the study. Additionally, compliance for retention of subjects in longitudinal studies in HDSS area can be significantly higher. Genetic history and pedigree can be of much importance in microbiome studies, which can be gathered easily in HDSS. Studies on first- and second-degree relatives, homogeneity and heterogeneity, endogamous (inbreeding) population, identical twins, and multi-generational studies are possible in HDSS.

Besides the points mentioned in this review, HDSS can assist in monitoring and tracking of new health threats, such as emerging and re-emerging infectious disease and drug resistance and alert the health community to prepare a response. A key advantage of HDSS over traditional cross-sectional studies is their ability to measure trends over time and, therefore, to provide a clearer picture of demographic and health developments. This approach is useful in characterizing populations at the community level and provides important information for health planning and intervention.

Health and demographic surveillance system sites have already shown its potential to conduct many epidemiological studies in chronic diseases such as hypertension, diabetes, and cancer (3, 4). Many HDSS sites are now also involved in genomic research and developing their health-related genomic research capacity through the Human Heredity and Health in Africa (H3Africa) consortium primarily, in Africa. In this way, this consortium brings genomic dimension to longitudinal population-based studies (66, 67). Developing such consortium for microbiome studies may add value for epidemiological and microbiome studies at HDSS sites.

Human microbiome research is relatively new and promising field, which requires an expensive infrastructure and expertise. HDSS sites can evolve and build capacity through a partnership with local, national, and international advanced institutions to enhance knowledge generation apart from the traditional observational, epidemiological, and interventional studies to systematic studies aimed at understanding the molecular mechanisms of the disease. Collaboration at national and international level can benefit both HDSS and human microbiome research.

## Future Directions for Lung Microbiome Research

In the past decade, sequencing-based techniques have generated abundant data, which has associated microbes with various respiratory health conditions including asthma and COPD. However, there is a paucity of direct functional and mechanistic data, which hinders clinical applications. Additionally, there are inconsistencies in the results, which can be correlated with various perplexing factors and technical challenges. These can be overcome through longitudinal, clinical, and *in vitro* studies in a defined population with similar environmental conditions. Cross-sectional studies conducted till date were hampered by their inability to explain the large variation in bacterial communities between individual samples and across subjects. Microbiome alterations/manipulation has a potential in disease control, which can be achieved through microbiota transplantation, dietary and lifestyle modifications, targeted antibiotics, interventions through probiotics and prebiotics. As this science progresses, it can open new therapeutic options for respiratory diseases. As there are complex association/interactions between microbes and humans, it is essential to study organisms and their actual roles in disease progression. Studies on larger cohorts with comprehensive metadata will help in deciding the directions, especially for personalized medicine.

## Literature Survey

We have reviewed the work done until June 2017 by searching PubMed, Science Direct, HighwirePress, and Google Scholar. The keywords for the search were airway microbiome, airway microbiota, asthma, COPD, metagenomics, microbiome, microbiota, obstructive airway diseases, epidemiology, HDSS, and combinations thereof.

## Ethics Statement

This paper is part of literature search done for a larger study titled “Exploring the Human Oral and Nasal Microbiota in Healthy and Diseased Population—A Study of Rural Population in Pune, India,” which is approved by KEM Hospital Research Centre Ethics Committee. A study was conducted following good clinical practices of research in the human population.

## Author Contributions

DA: initial draft and rewriting of all drafts to finalization. RP: initial draft and review of drafts. DD: review of drafts and rewriting of all drafts to finalization. YS and SS: review of drafts. SJ: conceptualization and review of drafts.

## Conflict of Interest Statement

The authors declare that the research was conducted in the absence of any commercial or financial relationships that could be construed as a potential conflict of interest.
